# Book Reviews

**Published:** 1821-01

**Authors:** 


### CRITICAL ANALYSIS

OF

### RECENT PUBLICATIONS, IN THE DIFFERENT BRANCHES OF MEDICINE AND SURGERY.

"I would have men know, that, though I reprehend the easie passing over of the causes of things "by ascribing them to secret and hidden vertues and properties; (for this hath arrested and laid "asleepe all true enquiry and indications;) yet I doe not understand but that, in the practical "part of knowledge, much will be left to experience and probation, whereunto indication cannot "so fully reach: and this not only in *specie*, but in *individuo*. Yet it was well said, *Vere scire "esse per causas scire*."—BACON.

*An Historical and Practical Treatise on the Internal Use of Hydro-Cyanic (Prussic) Acid, in Pulmonary Consumption and other Diseases of the Chest, as well as in several Complaints attended by great Nervous Irritation or acute Pain: with full Directions for the Preparation and Administration of that Medicine; and a preliminary descriptive Account of the principal Diseases in which it has been employed, illustrated by numerous Cases.* By A. B. GRANVILLE, M.D. F.R.S. F.L.S. M.R.I. Physician in ordinary to his Royal Highness the Duke of Clarence; Member of the Royal College of Physicians in London; principal Physician to the Royal Infirmary for the Diseases of Children; Physician-Accoucheur to the Westminster General Dispensary, &c. 12mo. pp. 417. Second Edition, greatly enlarged. Longman and Co. 1820.

THE present edition, it is said in the Preface, "may be considered as a new work, and the fourth attempt which the author has made, in the course of five years, to establish the claims of a new and powerful remedy to the attention of the medical profession in this country. To the original facts and observations respecting this important subject contained in the first edition, consisting of less than one hundred pages, the author has been enabled to add, since the period of its publication and rapid circulation in 1819, a vast mass of information collected from various sources, rendering it incumbent upon him to new-model the work, and extend it to its present size. He trusts that, in so doing, he shall not be taxed with having unnecessarily swelled his *pamphlet* into a *volume;* and that, when reference shall have been made to the additional matter of practical utility contained in the present edition, the scope he had in view in publishing it will not be mistaken."

The success of the author's exertions will be best shown by the relation of the fact, that about eight or nine quarts of prussic acid for medicinal purposes have been sold at Apothecaries' Hall since April 1819; and Mr. Garden, the chemist, says that he has disposed of forty pints since the same period. This quantity, for a medicine which is administered, at most, in the dose of from sixteen to twenty-four drops in the twenty-four hours, must be regarded as very considerable. There are circumstances, which it is unnecessary for us to enumerate, that render it becoming in us, on this occasion, to withhold the expression of the eulogies which reviewers, for their own self-complacency and satisfaction, are accustomed to indulge in, in their critical exordia, when they consider them to be merited; but, if any book ever had the less justice done to its deserts from the want of such panegyrics, this treatise will not, if we are successful in our attempt to give the reader of this article any thing like an adequate idea of its contents: for, the number of interesting facts which it presents, the assiduity with which they have been collected, and the perspicuous and methodic manner in which they are related, need only be contemplated in order that they may obtain the tribute which is due to the author, and an acknowledgment of the claims of the work to the attention of medical practitioners. We proceed immediately to our analysis, without any further prelude, as any remarks which we may have to make respecting the value of the remedy which it is the object of this treatise to place in its proper point of view, will be most properly brought forward when we come to consider its particular application.

After some preliminary reflections, comprising an account of the scope and objects of the present work, the author treats of the chemical history of prussic acid : this, being a matter of interesting curiosity, rather than of practical importance to strictly medical readers, we may pass over without adducing from it any more than an account of the constituents of this acid, as they have been determined by Mr. GAY-LUSSAC. According to this chemist, it is formed of carbon and azote, acidified by hydrogen. To the peculiar gaseous compound of carbon and azote, the base of the acid, he has given the name of *cyanogen*, (from *kyanos*, blue, and *gennao*, to produce,) which he has found to be composed of 46·19 parts of carbon, and 53·81 of azote, in 100; and the *hydro-cyanic acid*, of those same proportions of carbon and azote, with the addition of 3·90 of hydrogen : so that the acid is composed of equal volumes of cyanogen and hydrogen. This combination is, however, gaseous ; the liquid commonly known by the name of prussic acid, is a solution of this gas in *water*. Mr. H. C. JENNINGS (the inventor of the mariner's compass which is safe from magnetic influence of the ship in which it is placed,) seems to be the only chemist who has obtained pure liquid prussic acid, by means of immense pressure, as we stated in a former Number of this Journal, (November 1818.)

The second section of this work treats of the modes of preparing the hydro-cyanic acid for medicinal purposes. Those which have been hitherto recommended, are the processes of SCHEELE, as improved by LA PLANCHE; and of VAUQUELIN: each of which, Dr. Granville says, is perfectly good for medicinal purposes. That of Vauquelin appears to be most readily practicable by persons not well versed in chemical operations: t is as follows.

"Into a solution consisting of two ounces of cyanuret of mercury and sixteen ounces of water, pass as much sulphuretted hydrogen-gas as will serve to decompose the salt, leaving an excess of the gas. Filter the liquor to separate the sulphuret of mercury formed, and treat the filtered liquor with an excess of subcarbonate of lead. Shake the bottle until the excess of sulphuretted hydrogen be absorbed. Filter once more, and the remaining liquor will be diluted hydro-cyanic acid, of a proper strength for medicinal purposes."

The following is the formula which is employed at Apothecaries' Hall, and which was supplied by Mr. BRANDE.

"Prussiate of mercury (cyanuret), ℔j; muriatic (hydrochloric) acid, ℔j; water, ℔v. Draw off four pints, and rectify through chalk."

We regret that we cannot supply our readers with a point of information that they must here desire,—that is to say, an account of the precise proportions, in regard to strength, of the two preparations just described. It is the former to which Dr. GRANVILLE alludes when speaking of the doses, &c. in the course of this work; but no great inconvenience will be experienced by those who may choose to employ the preparation from Apothecaries' Hall, as the latter does not vary considerably from being double the strength of that obtained by the former, or Vauquelin's, process.

Some observations ensue on the presence of prussic acid in animal substances, when a few facts are noticed which have been thought to show that it has been found in the human body, under certain conditions, during life, though it does not naturally exist in animal substances in its proper form, but only in its elements. In several vegetable matters it exists naturally; as in bitter almonds, and various kernels having a similar taste to them; peach-flowers; peach and nectarine leaves; the cherry-laurel, (lauro-cerasus); the bark of the prunus padus; uva ursi, &c.: the whole of which possess the odour, as well as the taste, which is so strongly manifested by prussic acid.

The fifth section treats of the physiological experiments made with hydro-cyanic acid. Of the highly deleterious effects of this substance on animal bodies, when administered in certain quantities, every medical reader must be well-informed; and it cannot be unknown to them that fatal consequences have not unfrequently ensued from the use of laurel-water, laurel-leaves in custards, &c. cherry-brandy, noyeau, ratifie flavoured with kernels, and even from bitter almonds when eaten in considerable quantities. The colleges of London and Edinburgh have even deemed it prudent to reject the old black-cherry stone water of the shops, (formerly so favourite a remedy with the people in convulsive affections in children, and especially in those attending the eruption of small-pox,) from their Pharmacopœias, because of the danger attendant on the popular use of a medicine containing prussic acid. It was the striking similarity of certain qualities of taste and odour possessed in common by all those substances with the same qualities in the prussic acid, that led, by analogy, to the use of the latter as a medicine. Cherry-water and laurel-water had been for some years commonly used as remedies, the latter especially in Italy, when the prussic acid was first administered. Chemistry has since shown that the vegetable substances above enumerated contain prussic acid.

Several physiologists have endeavoured to ascertain the precise way in which the system is affected by prussic acid: that is to say, whether that substance acts by producing excitement or asthenia, what part of the body is especially and primarily affected by it; and how death is produced, when it acts as a poison: but none of these questions has been satisfactorily explained. Some have supposed, in the first place, that it acts as a direct sedative; but Messrs. DUVIGNAN and PARENT say, "we feel convinced that the prussic acid acts immediately on the nervous system, of which it excites the action in a manner very prompt, but transient;"* which seems more probable than the former opinion, for it is hardly possible to conceive that there is in nature a *direct* and *positive* sedative to an animal body. The notion of MANZONI and FANZAGO respecting its agency, is not unworthy of attention: they think that it acts as a stimulant to the part to which it is applied, whilst its influence on the rest of the system is sedative. We have pretty good evidence that a blister often acts in this way, by concentrating vital action in one particular spot; and it is not unreasonable to suppose that other stimulants may act in a similar manner. With respect to the parts of the body especially acted on by prussic acid, the opinion of MAGENDIE seems to be most probable: he thinks that it lessens, suspends, or annihilates, (according to the quantities in which it is administered,) the functions of *animal life* (of BICHAT), without acting directly

* American Medical Recorder, vol. ii. p. 464.

on those of nutritive or organic life. The latter become extinguished when the acid is administered in a large dose; but this effect equally ensues from certain degrees of injury to the organs of animal life, in whatever way inflicted, and when it is clearly evident that the deleterious agent has acted *immediately* on those organs alone. But, though we may, as physiologists, feel very desirous to have these questions decided, we may be pretty well satisfied, as medical practitioners, with the knowledge of the facts comprised in the following observations of the author.

"The prussic acid, when administered to a patient labouring under a disease of vascular or other excitement, appears to exert an immediate influence on the nervous system; it gradually diminishes irritability, checks a too-rapid circulation, and calms many of the symptoms of fever. If a dry cough be present, it promotes expectoration in the first instance, and subsequently stops the cough itself. The spirits, before exalted, soon feel the quieting impression of the acid; they become subdued; the speech, the countenance, even the expression of the eyes, assume a character of unusual meekness: there is a relief from pain and actual suffering; sleep comes on undisturbed, respiration is soft, and the pulse more tranquil than at any other period of the complaint, having lost the throbbing heat of irritation. In some few cases, these sedative effects are so much more considerable, that the patient expresses himself as if only '*half alive.*' On those occasions, there is an *apparent* entire prostration of strength, great lowness of spirits, and unwillingness to move, speak, or take food; (relative) life seems (almost) suspended, yet the head and mind remain clear and intelligent; there is a total absence of pain; neither does the patient complain of any symptom of local or general irritation; the heat of the skin is natural, and the pulse, in the midst of this dead suspense, continues its course steadily and quietly. This state of things lasts from twelve to twenty-four hours, when it ceases, and every organ is gradually restored to its former integrity of function. It should, however, be borne in mind, that such instances of great depression are extremely rare; and indeed seldom occur except where the dose has been too large, or the acid has been injudiciously administered; or from some peculiarity of constitution."

We pass over, for the present, some general observations on the efficacy of this medicine in relieving cough from various causes, hectic fever, asthma, chronic inflammation in various organs, pneumonia, pleurisy, menorrhagia and dysmenorrhœa, hemoptysis, and some nervous diseases,—to the section on the poisonous effects of the prussic acid, and the means of opposing them. We shall hereafter have occasion to notice the most important observations relating to the subjects previously enumerated.

Respecting the poisonous effects of the prussic acid, we shall only notice some observations made by Mr. COULLON regarding himself, and by Prof. HUFELAND, for the purpose of showing the different effects of different doses: though we suppose almost every medical practitioner must be familiar with the history of observations of this kind.

Mr. Coullon says, that, on taking an increased quantity of diluted prussic acid, which in the dose of twenty or thirty drops had not produced any considerable effect, he found the liquid to possess an extremely bitter taste; an increased secretion of saliva took place, and nausea in a slight degree occurred two or three times. The pulse became more frequent than ordinary, but returned to its natural standard in less than two hours. He experienced, for a few minutes, a sense of weight, and a slight pain, in the head about the forehead. Oppression about the chest, alternating with a slight pulsating pain, was also felt for about six hours. Prof. Hufeland relates the case of a robust and healthy man, aged 36 years, who, when about to be seized as a thief by the police officers, took a small phial containing a strong solution of prussic acid in alcohol from his pocket, the greater part of which he swallowed. He staggered a few steps, then sunk on his knees, and expired without appearing to suffer pain. Not the slightest trace of respiration or pulse could be discerned. A few minutes afterwards, a single and violent inspiration took place, which was repeated in about two minutes. The extremities were cold, the breast and abdomen still warm, the eye-lids were half open, and the eyes prominent and glistening: the face was not distorted, but rather like that of a person asleep. The corpse exhaled a strong smell of bitter almonds.

Dr. Granville says, that his own examinations of animals poisoned by prussic acid developed no appearances of organic lesion after death; and he says also that the muscles are not excitable by galvanism, when the dose has been very considerable. ORFILA seems to have remarked nothing apparently attributable to the acid in such circumstances, but congestion of blood in the veins and vacuity of the arteries, which is observed in cases of sudden death from several and diverse causes. He however says, that the muscles are excitable by galvanism for some time after apparent death. The blood seems generally to have remained fluid. None of the appearances in the bodies of animals poisoned by prussic acid, are peculiar to this state. The odour they exhale like that of bitter almonds, will however indicate the probability of its having been the cause of death. The most remarkable circumstance in the examination of bodies poisoned by prussic acid, when in a state not much diluted, is that it cannot be detected in the stomach, although the animal had died almost instantly after having swallowed it. It appears to be absorbed with great rapidity. We recollect having remarked that every part of the body of a dog manifested the odour of the acid for some days after its death, though it had been killed by the application of the poison (in a very concentrated state) to the nose.

The best antidotes to the deleterious effects of prussic acid, are diffusible stimulants. For the reasons above mentioned, any attempts to obviate them by chemical decomposition of the poison in the stomach must prove unavailing. Dr. Granville says, "Hot brandy and water, with, perhaps, some liquid ammonia,—or the latter in combination with camphorated spirit, properly, though sparingly, diluted,—or oil of turpentine, are, of all the means employed, by far the best and most effective." CHAUSSIER recommends a coffee-spoonful of oil of turpentine in a cup of strong coffee every half hour, for three or four times.

The ninth section treats, in a particular manner, of the history of the use of prussic acid as a medicine. As this is a subject of curiosity rather than practical utility, we shall immediately pass on to the second part of the work, which treats of the cases in which the prussic acid has been administered as a medicine. The first in order are cases of "consumption." On this subject the author says, in some preliminary general remarks, "Although it may yet appear, and I confess to me it is, problematical, whether a case of consumption, far advanced in the last stage of its melancholy career, has yet been cured in a decided manner by the prussic acid. There is no doubt that in hundreds of other cases, where the disease had not yet committed great ravages on the lungs and the system generally, that medicine has proved the means of arresting the progress of the complaint, and averting the impending fate of the patient." So far the author seems to be borne out by the evidence he adduces in this work; and it is such as will, probably, leads us in our own practice to supersede, in many instances, digitalis, tartar emetic, and ipecacuanha, by the prussic acid: but, that this or any other medicine will prove the means of *cure* of *tubercular* phthisis, is a thing which does not come within the circle which we regard as the boundary of probabilities.

There seems to be more of the humoral pathology comprised in Dr. Granville's opinions than is generally admitted at the present day. On speaking of the several species of consumption, (in the manner of a slight sketch only, as a description of diseases, beyond the principal traits which characterize them, does not make one of his objects,) he states one of them to be "owing to a vitiated state of the animal fluids, no matter from what cause, whether scrofulous, scorbutic, syphilitic, or otherwise." This species, he says, "includes what has been called the tubercular and strumous consumption." Alluding to the

*incipient* stage of this disease, Dr. Granville says, "If the prussic acid be given during this stage, even where a constitutional tendency to this dreadful malady exists, and before the suppurating process of the tubercles be formed, a perfect recovery may be expected. This is well illustrated by the following cases." The cases alluded to are ten in number. As our scepticism respecting such powers in this remedy, and the improper prejudice which is, perhaps, founded on that scepticism, may prevent our seeing in the cases referred to such evidence as the author considers them to present, we deem it prudent to transcribe some of the cases in question, rather than to attempt to give an abstract of them, in which we might, perhaps, omit the signs which are really characteristic of *tubercular* phthisis. Our limits will not permit us to transcribe the whole series; we shall, therefore, select those which occurred to Dr. Granville's own observation. We shall, by choosing these, be most likely to do justice to his statements; and we cannot help, besides, observing, that most of the others are remarkable for the extremely imperfect and unsatisfactory manner in which they are detailed.

"Case IV.—Master Blackwell, aged ten years, was sent to sea as a midshipman, in hopes, chiefly, of his overcoming a certain predisposition, as it was then supposed, to strumous consumption. For some time after the constitution seemed to improve; but about three years ago, having spent a very severe winter on the Newfoundland station, the symptoms of pectoral disease became so pressing, that, on the recommendation of the medical officers, and indeed of Captain Buchan himself, his commanding officer, he was sent home. On his arrival in England, I was requested by the late much-lamented Sir W. Farquhar, then ill, to visit him; and my report to that eminent physician was altogether unfavourable. Every mean usually employed in such cases was resorted to, in the first instance, to no purpose. The boy was wasting daily; the cough and night-sweats had manifested themselves in a decided manner. There was no expectoration approaching to purulency; nor was the abdomen enlarged or distended. The bowels yielded to proper medicines, and the secretions appeared healthy. The pulse 125 or 130; the skin dry; the respiration difficult; the cheeks flushed. The prussic acid was administered with carbonate of potash. In about three days the disorder seemed arrested. He continued the same medicine another fortnight, and every symptom, except debility, disappeared. He is now in excellent health, and has been so for upwards of two years. The almost instantaneous good effect which the acid seemed to have, from the first, on the cough, induced the mother, who had been troubled with that symptom during the whole winter, to take, without my knowledge, the same mixture with the acid which I had prescribed for her son; and it was only after her cough had wholly subsided that she acquainted me of the step she had taken, though she knew not of the mixture being any thing else than a common cough-mixture.

"Case V.—A servant of Mrs. E——'s, of Upper Seymour-street, was sent to me for advice, in the month of May, 1819, in consequence of a complaint in the chest, which had been, more or less, troublesome for several winters, but which had become more urgent at the time of his consulting me. He was then in the twenty-second year of his age, tall, slender, of a very fair and delicate complexion, with blue eyes, light hair, white pearly teeth, long pale fingers, &c. He complained of pain immediately under the sternum, a little to the right; coughed occasionally, without any expectoration; breathed with difficulty, and as if heavily oppressed in so doing. The pulse was frequent, small, irregular. There was a clammy moisture on the skin; yet the palms of his hands were burning with heat; his cheeks were marked with spherical flushes, which contrasted singularly with the bright-blue tint shining through a pearly-white skin, about and under the eyes. The tongue was of a purplish colour. The appetite good, eating usually three meals a-day; but the body wasted notwithstanding. The bowels rather relaxed; the abdomen soft, and of a natural size. Being questioned about any chilliness after meals, he answered in the affirmative. The patient had been occasionally attended by the family apothecary, and had been twice bled, upon which every symptom seemed to grow worse; until, at last, his mistress resolved to send him to me for further advice. Considering his case to be one of incipient strumous consumption, I prescribed the acid, which the patient himself used to procure at Mr. Garden's in Oxford-street. During the first week the cough subsided, as well as the perspiration, but the pulse continued frequent and small. I then gave, alternately with the acid, a mixture with myrrh and carbonate of potash, which seemed to strengthen him; and, during the five weeks that the patient continued to call on me, I could plainly perceive the good effects produced by the medicine. At the end of this time, the cough had entirely left him; the pulse had been lowered to about 80; and he considered himself so much better that he discontinued his visits. I understand that he applied for some time longer to the chemist for the acid, and that, when he had last been there, he appeared in perfect health.

"Case VII.—Miss C—— had from her childhood exhibited signs of scrofula, though born of healthy parents. The glands of the neck; occasionally, those of the groins; and, in one instance, those under the left arm had become swelled, painful, hard, but had never suppurated. On some of these occasions there was a slight cough present, which was attributed either to teething or to worms. Her appearance, however, denoted perfect health, and no uneasiness was felt respecting her. Towards the critical period of fifteen, after no inconsiderable sufferings, the glandular swellings disappeared almost suddenly; and in a few months afterwards menstruation became fully established. The young lady was now considered as perfectly healthy, and no farther attention was paid to her. As soon, however, as the formalities of etiquette required her presence in the gay world, and she was surrendered to the caprice of fashion, it was discovered that she could not well bear the fatigues of the night, and that neither the prolonged hour of rising on the following days, nor the measured and choice diet, nor the drive in the open landau, were sufficient to stay the wasting effects which such a mode of life had visibly produced. A *slight* cough again made its appearance, accompanied with a *slight* pain in the chest, and a *slight* oppression in breathing. She was *slightly* affected when walking fast, or ascending the stairs; and she felt, though *slightly*, every variation of the atmosphere. The symptom which first alarmed the parents, was emaciation; and this seemed to proceed so fast, that Miss C——, from being an object of pride, became soon an object of pity and grief, to her friends. A physician was called in, who thought that the young lady suffered from diabetes, and ordered a treatment accordingly. A second was soon afterwards consulted, whose more particular study was supposed to be the unravelling of mysterious complaints, and particularly of the chest; for by this time a disease of that part was strongly suspected by all. His opinion confirmed the fears of the friends, but his treatment did not allay them. In the month of June 1819,—that is, eighteen months from the first time of taking the alarm, the patient was supposed to be in a consumptive state, and under these circumstances I was requested to give my opinion. Mine could not but be in unison with that of the medical gentleman who preceded me; and I strongly recommended the use of the prussic acid. Indeed, my opinion had been asked specifically as to whether that medicine was admissible in the case of Miss C——. To describe all the symptoms under which this young lady laboured at the time of her first beginning the use of the acid, would be to repeat almost every symptom which has been before mentioned in the outset of this section. Suffice it to say, that she was considered as being in the first stage of tubercular consumption. I visited the patient occasionally from June till August, and derived great satisfaction at the amendment which I thought I could perceive in her general health. My subsequent absence from England prevented my following the case through its various stages; but, on my return from the continent in November, I had the pleasure of learning, by letter, that my patient, then in the country, had, during the autumn, lost her cough and oppression on the chest; that she had gained a degree of *embonpoint*; and that, at the approach of the bleak season, she had not experienced, as she had done the preceding years, the ill effects of cold and wet weather. The acid had been discontinued for three months, and resumed in February of the current year; when, after a fortnight, it was altogether abandoned, the young lady being in all respects free from complaint. In this case the acid never produced either sickness or dizziness. It seemed to act gently on the bowels for a few days, and not longer; and was administered, both when it was first taken and when it was resumed, for the first week, in doses of one drop every two hours in a common camphor mixture. This mode of prescribing the acid I have found to be preferable to that of limiting the use of the medicine to three times a-day; when, if the whole quantity be pushed, from necessity, to eighteen or twenty drops, the patient is forced to take six of them at a time; a dose which seldom fails to produce sickness, and seems to irritate rather than to act as a sedative.

"Case VIII.—A lady above sixty years of age, the grandmother of several children, had been troubled for several years with a difficulty of breathing and cough, which, on reaching the period of menespausia, became very troublesome, and excited some alarm. In the early part of her life she had enjoyed excellent health, but signs of an unfavourable predisposition to glandular obstructions and enlargements had manifested themselves at various periods, and have since remained stationary. Several glandular swellings were shown to me on the second visit, in February 1820, occupying various parts of the body, but particularly the neck, the bosom, and each side of the chest. Remedies of all descriptions had been resorted to without effect: some indeed, which had been immediately directed to the removal of the glandular swellings, proved pregnant with mischief, and were soon abandoned. With the cough there was dyspnœa, and a scanty thick expectoration. She complained of fever at times; and her pulse, on my first seeing her, was full, hard, and above a hundred strokes in a minute. The skin dry; the tongue also dry, roughish, and of a pale tint. Slept uneasily, and at interrupted intervals. Her bowels had for a long time been confined, so as to render the use of daily medicines necessary. There was no decided pain in any part of the chest; but much uneasiness, impossibility of making a deep inspiration, palpitations and flutterings, with a kind of spasmodic attack at times, which rendered her existence truly miserable. An opinion had long before been given, that this lady was labouring under some tubercular formation in the lungs,—an opinion which external appearances, and the symptoms already described, seemed greatly to uphold. Occasionally the cough, expectoration, fever, and morning perspiration, with a hard pulse, would gradually become so alarming as to induce the attendant practitioner to prescribe bleeding. This operation was about to be again performed when I saw the patient, and it was determined, in preference, to give the prussic acid a trial. From the notes of my first visit, I find that her pulse was hard and frequent; that she had had several attacks of chilliness and fever; that she slept ill, and expectorated a sort of grumous yellow and greenish matter, which, when thrown into cold water, went to the bottom; and, when acted upon by boiling water, did not shrink, so as to appear smaller in its volume.* I ordered the prussic acid in the dose of ten drops for the twenty-four hours, powders composed of tartarized antimony and supertartrate of potash, and some aperient pills.

"The relief she obtained from this treatment became evident in a very few days: the cough soon afterwards ceased altogether, and with it the expectoration. I first saw my patient on the 15th of March, and by the 30th I discontinued my visits. She is now in her former usual state of health, and acknowledges the great benefit she has derived from the medicine in question."

In the *confirmed* stage of consumption, "when the suppurative stage of the tubercles has fairly begun," says the author,

* I beg to suggest this among many other distinctions between pure mucus and decidedly formed pus.

"the hopes of recovery, from the effects of the prussic acid, become every day more faint, until at last nothing but palliative effects, and those of short duration only, can be expected. Still there can be no doubt that, even in the advanced period of this complaint, alleviation of symptoms, improvement, and even recovery, can, in some few instances, be looked for; since cases have occurred where the life of a patient, under the most unfavourable circumstances, has been redeemed by the action of the medicine in question; and in some other cases by other medicines also, as Dr. Laennec has proved in his recent and excellent work on Pectoral Diseases."

How far the cases noticed by Dr. Laennec were benefited by any medicines that might have been employed, is a matter of doubt; but, that much alleviation has ensued from the use of prussic acid in the latter stage of tubercular phthisis, is abundantly shown by the cases adduced by Dr. Granville; and this alleviation seems to have been much greater and more frequent than what has been derived from any other medicine. The first case of this series is, indeed, related by Dr. Magendie as an instance of the *cure* of tubercular phthisis in its *second* stage: the degree of confidence in this must depend on the degree of credit that may be thought to be due to Dr. Magendie's *assertions* that it was such a disease; for he has not, it would appear, thought it necessary to satisfy the public that such was probably the case, by any historical evidence. We shall transcribe it as it is detailed by Dr. Granville.

"A lady from Lyons, now residing in Paris, of a constitution eminently bilious, after having experienced several misfortunes, was, in 1814, attacked by all the symptoms which characterize phthisis in its first stage. Circumstances not allowing her to attend to her health, she neglected it, until the month of January 1815, when, the disease having made great progress, she consulted Dr. Magendie. He found her labouring under all the symptoms of the second stage of tuberculous consumption, with a cough returning incessantly, and a slow continued fever preying upon her and undermining her existence. The prussic acid was recommended, and taken in the dose of from six to ten drops in twenty-four hours, diluted. The medicine was continued for about two months. From the first day the cough diminished, the patient slept; and, without increasing the dose beyond ten drops in the twenty-four hours, all the symptoms of the disease disappeared; the breathing became natural, the cough, expectoration, and sweats, ceased. In short, the lady was perfectly cured, and has never since experienced any symptoms which indicate the least disposition to a relapse. Her lungs, only, have become very sensible to the influence of atmospheric variation."

On alluding to the foregoing case in his pamphlet published in 1819, Dr. Magendie says that the subject of it continues to evince perfect health.

A case follows by Dr. Granville, in which it is satisfactorily evident that consumption in its latter stage, in a young man, "though not cured, was so far palliated as to render life tolerably comfortable." How long this state of amendment continued, is not mentioned. This case is succeeded by that of a young lady, who was considered by her medical attendants to have "phthisis pulmonalis;" and Dr. Granville says, "a description of all her symptoms was forwarded [to him] in confirmation of that opinion." Those stated to be present by Dr. Granville are, pain in the chest, cough, restlessness at night, and great prostration of strength. We must rely on the accuracy of his observation and judgment, that the rest of the evidence necessary to characterize phthisis pulmonalis was present. This patient rapidly recovered so far under the use of the prussic acid as to be able to leave this country for Malaga, (which place Dr. Granville strongly recommends as a residence for consumptive patients,) though a few weeks before she took the acid "she was threatened with imminent dissolution;" and she has recently returned to England, "to all appearance in excellent health."

We must not omit to notice the remark with which Dr. Granville concludes the history of this case, lest our former transcriptions should convey erroneous ideas of the views he entertains of the powers of the remedy under consideration. Dr. Granville says, "Where there is disorganization of the lungs, no cure can be expected; but life may be prolonged, and rendered comparatively comfortable:" and he elsewhere (pages 241 and 242,) adduces similar remarks; showing his disposition to confine his inferences to what is strictly warranted by the evidence of observed facts.

After two other cases, extraneous to the author's own observation, in which some alleviation appears to have resulted from the use of the prussic acid, Dr. Granville takes occasion to make some remarks on the occurrence of vomiting from the influence of this medicine. He says,

"When the prussic acid produces nausea, or even vomiting, (and it is the same with regard to dizziness, which it will induce in some few individuals,) those symptoms take place on the first or second day after the exhibition of the medicine, unless indeed the dose be greatly augmented; and in such cases I strongly recommend that it should be abandoned immediately, for there is no chance of its ever again agreeing with the patient. Of about three or four hundred cases of the exhibition of this medicine, to which my personal experience extends, five or six have occurred in which the acid, evidently from a particular idiosyncrasy, produced sickness at the stomach on the first day it was taken; and in no other was any nausea excited, when it had, to all appearance, agreed with the stomach for the first week. This peculiar effect of the prussic acid on the stomach of some few individuals, *on its first exhibition*, is wholly independent of the quantity taken."

Two other cases, which occurred to the observation of the author, are next related; in one of which, especially, a degree of alleviation was produced by the prussic acid that we should not expect to witness from any other medicine hitherto employed. A case is then related by Mr. RUDLAND, of Dartmouth, which seems to us to have been chronic bronchitis, consequent on acute bronchitis, and not "phthisis;" but there was a family predisposition to consumption, and the symptoms in this patient (the mother of several children) were very alarming, and such as often precede real tubercular phthisis consequent on bronchial inflammation. This patient entirely recovered her health under the use of the prussic acid, after, as it is said, "the most approved therapeutical means were adopted and rigidly followed for many weeks, without any obvious benefit."

Several other analogous cases are related or referred to; and then the author treats of its use in another species of consumption, which is that "subsequent to catarrh, pneumonia, pleurisy, bronchitis, and hæmoptysis." The diagnostics of this, in contradistinction to those of *idiopathic** tubercular consumption are first discussed; and then several cases illustrative of the powers of the prussic acid in the species of consumption arising from the causes just enumerated, are detailed. This is the part of the work which we have perused with the greatest degree of satisfaction; and we feel convinced that, in its application to the cases of the kind here alluded to, the prussic acid will prove a highly valuable addition to the materia medica; and with such impressions we very earnestly recommend the cases here related by Dr. Granville to the perusal of our readers. Several instances are adduced of the recovery of perfect health from states which were considered as hopeless by practitioners of eminent talents and very extensive experience. Besides the result of his own experience, Dr. Granville here, and elsewhere, brings forward that of others; amongst which is a very interesting account of a case of "phthisis trachealis" by Mr. TODD THOMSON, in which the efficacy of prussic is shown in a very favourable point of view.

Dr. Granville considers, in a distinct manner, consumption "occurring during pregnancy, or immediately after parturition, also after long suckling, or at the period of ablaction." He thinks, with some former authors, that "pregnant women

* We designate it thus to distinguish it from the tubercular consumption which seems to ensue from bronchitis as an effect of the latter affection, as we showed in our exposition of the doctrine of Dr. BROUSSAIS.

are sometimes attacked with phthisis from some cause originating in the peculiarity of this situation;" but he has not thought it conformable with his object in this work, to bring forward his arguments for this opinion. The results from the use of the prussic acid in these cases are detailed; but, as they are analogous to those derived from this medicine in the other forms of consumption, we need not stop to particularize them.

The last species of consumption enumerated by the author, is "from a single abscess or vomica, the effect of accidental inflammation of the pulmonic texture, or of the membranes, in consequence of local injury." He states that he has hitherto had no opportunities of trying the prussic acid in this variety of pulmonic affection. According to Dr. Laennec, abscesses of this kind are of but very rare occurrence, as we remarked in our review of his work on Diseases of the Thoracic Organs.

The second section of this part of the work treats of the efficacy of prussic acid in "pneumonia, pleurisy, and other inflammatory complaints."

That so powerful a sedative, and one so rapid and almost constant in producing its effects, as the prussic acid, should be very efficacious in those affections, might be readily preconceived: the author has not, therefore, thought it necessary to give many cases of the kind alluded to in detail. The indications for the application of the remedy must, also, be sufficiently obvious, and do not materially vary from those which have commonly led to the use of tartar-emetic, digitalis, and colchicum, on analogous occasions; like which remedies, too, the prussic acid seems to be eminently qualified to obviate the practice of excessive blood-letting in many instances, when, without them, it might be thought necessary. Distilled laurel-water, as we have frequently shown in late Numbers of this Journal, is very commonly employed in Italy in consonance with those views. The foregoing remarks are equally well applicable to the subjects of several of the third section, which treats of the use of prussic acid in "catarrh" and "spasmodic cough:" the fourth, on its employment in "hectic—and sympathetic cough;" and the fifth, in whooping-cough. For the details of the evidence on these points, we must refer the reader to the work: we shall only remark, that, as far as regards the two former series, the evidence is such as shows that the prussic acid may very well supersede, on many occasions, any one remedy previously employed. This seems to be especially the case in respect to severe catarrh, as it appears epidemically at certain periods. On speaking of whooping-cough, the author draws a picture of the conduct of some practitioners in public institutions, which we trust is not strictly applicable to any one. Many men may think that they have, and they undoubtedly have, treated whooping-cough successfully by antiphlogistic means; and it is as unjust to attribute to "high-mettled fancy" their use and praises of such measures, as it would be to attribute to such qualities the author's praises of the prussic acid, which would itself never have been employed, if the remark that those who use it "must certainly have forgot their *Cullen*, or know more than that celebrated physician," were justifiable. The sentiments we have just transcribed cannot, however, have been intended to be seriously expressed; for they inculcate nothing less than that no man can hope to be better informed in pathology than Cullen was, or treat whooping-cough better than he did. Dr. Granville says,

"I will, however, state, once for all, that the whooping-cough, in itself, is *never* an inflammatory disease,—for no traces of inflammation have been found in the respiratory organs of those who have fallen victims to it; and that, when the complaint has been very violent, and has lasted a great length of time, and then only, tokens of inflammation have been found in the brain, as the result of strong and often-repeated spasms of the organs of respiration, producing a great determination of blood to the head.*"

Without information of the stages of the disease in which the dissections mentioned in the note were made, the evidence of the want of signs of inflammation of the respiratory organs is hardly worthy of attention. If it is to be argued that whooping-cough is not originally an inflammatory affection, because no signs of inflammation are to be found (supposing the anatomical examinations to have been made in an accurate and sufficient manner) after death, in certain stages of the disease; it might just as well be argued that a gall-stone, passing through the choledochus, has not been the cause of the pain and vomiting which the patient thus circumstanced has experienced, because the stone is not to be found in the duct after death. It would seem, too, that the accuracy of the observation of the persons alluded to in the note just transcribed is not very satisfactorily shown, when they say that "the wind-pipe was found constantly *healthy*, although lined with a frothy, *blackish*, and *adhesive* mucus." We never yet saw an instance where the body of a child who had died in the early stages of whooping-cough was

* "In the first volume of the *Memoire Scientifiche e Letterarie dell' Ateneo di Treviso*, published in 1817, there is a paper containing the result of extensive pathological inquiries, made by several physicians and surgeons of that town, into the nature and seat of various complaints. On the subject of whooping-cough, which had been epidemical in 1816 at Treviso, a table of twenty-three anatomical examinations of children after death is given; from which it appears, that in all of them there were more or less signs of turgidity of the blood-vessels of the head, or serous effusion; that no symptoms of disease occured in the chest, except in a few individuals, who presented an incipient phlogosis, or plethora, or serous effusion in that cavity. The wind-pipe was found constantly healthy, although lined with a frothy, blackish, and adhesive mucus."

examined, without signs of inflammation in the larynx or some part of the trachea being manifest; and many practitioners of our acquaintance give testimony of a similar kind. Whether the inflammation is essential to the disease, is at present, we think, a matter of doubt, not a point that can be decided by an absolute negative. It is possible that whooping-cough only destroys life when it produces some of the diseased states which other practitioners regard as essential to it, and that it is really, in itself, as Dr. Granville seems to think, "a spasmodic affection." That the prussic acid possesses great powers in curing it, appears to be well proved by the cases here brought forward, and we cannot neglect to congratulate the author on his successful and judicious application of the remedy to so severe and often fatal a disease. We shall transcribe the two series of cases first in order, (that we may not err by an improper choice,) for the purpose of exemplifying those remarks, and of showing, at the same time, the mode of administering the remedy.

"Two children of Major Fitzgerald fell ill with the whooping-cough, in June 1819, at some distance from town; and, having been early consulted respecting the best mode of treating them, I lost not a moment in suggesting the use of the prussic acid. I also requested to be informed of any change that might take place during its use, and of the apparent effect of that medicine on the children. On the 23d of the same month I received a letter, stating that, "on the eleventh day after taking the prussic acid, my little patients were almost recovered; that the youngest had ceased to cough the last three days; while the eldest coughed still, but a loose, easy, and common sort of cough; and that, although there was still a whoop accompanying it at night, it was but a feeble one.' 'The disorder,' the letter continued, 'was dying away, as they eat heartily, and the blackness under the eyes was quite gone, as well as the fever, langour, restlessness, &c.' I recommended a change of air, and this advice was followed almost immediately. The cough soon afterwards left the eldest girl also; and they both returned to their home quite recovered. A relapse, however, occurred in the latter place; and the prussic acid was again had recourse to, and continued until no trace of the complaint remained behind.

"Four young children, three boys and a girl, of Wm. Hamilton, Esq. were attacked, almost simultaneously, with the whooping-cough, in the month of May last; the two youngest with fever and symptoms of pyrexia, to which vomiting soon after succeeded. After opening the bowels with suitable medicines, and giving some cooling powders to those who had fever, I proceeded to administer the prussic acid to all, at first in an almond emulsion, and next in a camphorated mixture. The effect of the medicine was not the same in all four. One of the patients ceased to whoop almost immediately. The two next continued to cough for some time longer; and a fourth, the youngest but one, seemed scarcely to feel the influence of the medicine,—for he continued, even after the complete recovery of his brothers and sister, to cough, whoop, and vomit. His general health, too, appeared to suffer from the prolongation of the complaint. The girl was the first who got well; for at the end of ten days she was scarcely ever heard to cough, and then in the ordinary manner only of people affected with catarrh. She had no relapse. Three of her brothers got well next,—that is, within the third week of taking the acid; but a parotid and tonsillar swelling, with fever, occurred to one of them at the same time, which required the application of leeches, and the adoption of a brisk system of evacuation by the bowels. The prussic acid was continued notwithstanding; the patient taking a tea-spoonful of the mixture, containing about one drop of the acid, every three hours. At the end of six weeks his recovery also was complete."

A considerable extent of forcible evidence of the very successful agency of this remedy in the same disease, is also brought forward from other practitioners, by whom it has been communicated to the author.

Analogy, on the facts already noticed, led the author to employ the prussic acid in asthma; but hitherto, he says, he has had but few opportunities of applying it to this disease. When we consider the various causes from which asthma will arise, and how frequently it is a consequence of considerable organic lesion of some of the thoracic viscera, we cannot expect that this remedy will be very generally of much utility here; but it seems probable, as the author argues, that it may occasionally be beneficial, and three cases in which it has been so are detailed in this work.

Hæmorrhages—painful menstruation—and abortion, are other cases in which the prussic acid has been employed with well-marked, and in some cases, in all probability, with peculiar, advantages. We shall not dwell on the successful results here brought forward, lest we should lead some of our readers to suppose that we think this medicine may supersede free blood-letting, and revulsive measures, in the generality of cases of hemorrhage. They will best appreciate the evidence after perusing it in detail. There is not, however, much reason to fear that the generality of practitioners will become too enthusiastic and exclusive in the use of a new remedy, however powerful: they will regard it as a valuable addition to those already employed, not as one that is to supersede them on all occasions.

"Nervous diseases" and "affections of the stomach," are comprised together as the subjects of the eighth section. This commences with some good remarks on the affections ordinarily termed nervous; a great proportion of which, the author says, arise from some disorder of the stomach. But, he adds,

"Every complaint which we cannot well class with other well-known affections, we often attribute to the nerves; and, in styling a disease *nervous*, we are often using a mere name to denote, at one and the same time, a vast number of phenomena, definite in themselves,—such as tremor, fainting, palpitations, throbbing, flying pains, flatulency, indigestion, uneasy sensations, spasms, constipation, sudden startings, sighing, laughing, a lump in the throat, and the whole long train of ailments which are known to affect, in a more particular manner, the fine lady,—the irritable,—persons leading a sedentary life, especially authors, projectors, ambitious schemers, disappointed politicians, with many more restless and discontented beings."

Several cases are related of great disturbance of the nervous system from various apparent causes, which were obviously much benefited by the prussic acid; and, in regard to its efficacy in dyspepsia and some other affections of the stomach, we may add to the evidence brought forward by the author, that Dr. Broussais has satisfactorily traced them, as well as hypochondriasis, in a considerable proportion of cases, to chronic inflammation of the stomach.* We select the following case, which is related by Mr. Todd Thomson, as a good exemplification of the utility of the prussic acid in a severe form of the affection just designated.

"T. R——, esq. of a slender form and gouty diathesis, had long been afflicted with dyspepsia, attended with a peculiar hot sensation of the tongue, which was supposed to depend on acidity of the stomach. The remedies he had employed, and the regimen to which he had confined himself, for some time past, had materially improved the power of the digestive organs; so much so, that he declined the further use of medicines, and considered his health to be as good as it could be expected to be in an individual beyond the middle age of life. Notwithstanding this improvement, however, the heat of tongue still remained, when he was attacked with the epidemic catarrhal cough, already mentioned. He took the acid in doses of two minims repeated every second hour; and, with the cough, the heat of the tongue also rapidly abated, and altogether left him in less than four days. I have not heard that the latter symptom has returned.

"As the state of the stomach affects the tongue by sympathy, perhaps the unexpected effect of the acid in this instance may be ascribed to its reducing the morbid irritability of the secreting surface of the stomach, thereby enabling the juices of the organ to be more slowly secreted, and of a more healthy character. We know that opium and some other narcotics produce temporary relief in cardialgia, arising from acidity; but, after their first effect is over, the morbid irritability of the organ not only returns, but is augmented: if, therefore, the prussic acid produces a more permanent and equally-beneficial effect, its importance, as an adjunct to tonics in the treatment of dyspeptic affections, must be obvious."

A very interesting letter, by Mr. Thomson, ensues, on the

* See the exposition of the doctrine of M. Broussais in the late Numbers of this Journal.

general utility of the prussic acid, in which the testimony in its favour as "a remedy of the greatest value" is very decisive.

The author briefly hints at the benefits which have resulted from the topical application of the diluted prussic to various parts; but he defers, until he shall have had more experience of its effects, entering fully on this subject.

The therapeutical part of the work concludes with an account of two instances of "phthisis" "*cured*" by the prussic acid, which have occurred to the knowledge of the author since the former part of the book had passed the press. We shall transcribe the case which was seen by Dr. Granville himself; for we must acknowledge our inability to discern in it evidence of the existence of *tubercular* consumption, (which is the species to which the term *phthisis* is generally applied by pathologists in an exclusive manner, and is the only one that is commonly regarded as incurable by means well known to every practitioner.) By transcribing cases thus at length, we extend this article much beyond what we should do; but we know no other way of avoiding the danger of misrepresenting the contents of the work.

"Miss Munn, aged 17, of a nervous temperament, with dark hair and regular features, the daughter of healthy parents, and herself enjoying generally perfect health, was attacked, towards the latter end of April, 1820, with every symptom of catarrh, difficulty of breathing, pain in the chest, fever, and restlessness, capable of alarming her relations. Her mother, having occasion to see Mr. Clarke of Saville-row, obtained from that gentleman a prescription, in the use of which the patient persisted for some days, without any sensible amelioration of her complaint. She indeed became considerably worse at last; when, to the pain in the chest and slight febrile rigors, were added a considerable expectoration of thick purulent matter, with regular paroxysms of fever, profuse morning perspirations, head-ach, sickness, cough, and a general wasting of the body. In this state she was taken to Dr. Batty, who ordered her to be bled, and prescribed appropriate medicines, declaring her at the same time to be in a consumptive state, and therefore beyond the chance of recovery. To this decision the afflicted parents endeavoured to reconcile their own and the poor girl's feelings; while every succeeding week seemed to confirm the correctness of that physician's opinion. On the 26th of June, Miss M. was brought to me for advice, when she presented the following symptoms, which I copy from my note-book: A general emaciation to a very great degree; eyes sunk and without animation, marked by an under and broad streak of a leaden hue; nose pointed and sharp; the alæ playing strongly and quickly during respiration, which was heavy, difficult, and accompanied by a considerable wheezing noise. Soreness and a sense of perpetual tickling along the wind-pipe; pain deep-seated in both sides of the chest; great tenderness, on pressure at the pit of the stomach; dry and arid skin; foul tongue; hot breath; pulse 115, small and wiry; excessive debility; spirits much dejected, the patient bursting into tears at each question put to her on the subject of her complaint. The mother reported, moreover, that she sleeps for a few minutes only at a time, owing to an incessant hard cough; that she wakes in the morning bathed in sweat; expectorates a large quantity of thick grumous matter in the course of the day; that at night she is attacked by a regular paroxysm of fever, when she sinks on her bed powerless and exhausted, to rise next morning to a repetition of every symptom.

"This picture afforded nothing very promising. I concealed my impressions from the alarmed patient, and cheered both her and her mother with the prospect of some amelioration from the use of the hydro-cyanic acid, the nature of which medicine had, somehow or other, come to their knowledge. The form in which I prescribed it was that marked No. 7; and additional instructions were given respecting every other part of the treatment, which it is needless to repeat in this place, as they differed but little from what is usually recommended in similar circumstances. The beneficial effects of the acid became visible almost immediately. At the end of the first week, the cough, and consequently the expectoration, had diminished greatly; sleep became prolonged and refreshing; the morning perspirations ceased; the pulse was reduced to 100 beats in a minute, and assumed a healthier character. The dose of the acid, which had been limited to a drop and a half every three hours at first, was now increased to two, and she was desired to persist in it for another week. This she did with so much benefit, that the mother, considering her now as quite safe, discontinued the prussic acid, and merely attended to my instructions as to her general health. I saw the patient again in a fortnight, and the change which had then taken place in her appearance seemed to be so favourable, that I could scarcely believe it to have been the work of so short a period. I enjoined great care and quiet, and ordered some tonic medicine, contenting myself with hearing from her from time to time. Towards the end of September last, the accounts were that she was enjoying perfect health, and had done so for several weeks before. Of the truth of this assertion I had the means of satisfying myself in about a fortnight afterwards, when I found Miss M. free from every, even the most distant, symptom of complaint. It is proper to observe, that Miss M. has not yet menstruated, and that she is small of her age."

The following is the formula referred to in the foregoing history:

"℞. Mucilaginis acaciæ, *f.* ℥ iij; aquæ rosæ, *f.* ℥iiſs; syrupi capilli veneris, *f.* ℥ ij; acidi hydrocyanici med. *m.* xvi. F. M. L. A. *Cochleare unicum medium, si vis, singulis horis excipiatur.*"

The work concludes with a section on the "mode of prescribing the prussic acid," and a list of formulæ. The substances with which it may and may not be combined, are particularly discussed. It is not decomposed by mixture with any vegetable substances. It is decomposed by most of the oxides usually employed in medicine, particularly by those of mercury and antimony. It may be given in conjunction with the carbonates of potash and soda; "forming one of the most successful modes of prescribing it in cases of spasmodic and whooping cough; and supplying the means of administering it in fevers under the form of an equally pleasant and elegant preparation." We refer the reader to the work for the author's particular considerations on its pharmaceutical relations to the other substances of the materia medica in ordinary use; only remarking, that the *protoxide* of mercury (a preparation which GUIBOURT says cannot be obtained, and that what has been called a protoxide is a combination of deutoxide with metallic mercury finely divided,) is said by the author to be the only preparation of that mineral with which it may not be administered. It is of considerable importance in the administration of this medicine, Dr. Granville says, that the mixture containing it should be newly prepared every day, as it seems, on ordinary occasions, to suffer decomposition on being kept for a longer time than twenty-four hours.

---

*Illustrations of the Efficacy of Compression and Percussion in the Cure of Rheumatism, Sprains, and Debility of the Extremities.* By WILLIAM BALFOUR, M.D. Author of "Illustrations of the Power of Emetic Tartar in the Cure of Fever, Inflammation, and Asthma, and in preventing Consumption and Apoplexy." 8vo. pp. 36. Hill and Co. Edinburgh; and Longman and Co. London. 1820.

THE fate of the practice of "compression and percussion" in the treatment of rheumatism, exemplifies, in a very forcible manner, the difficulties which attend the bringing a new remedy into general usage, when that remedy differs in a considerable degree in its nature from the means which may have been previously employed. Dr. BALFOUR, and some others who have adopted his practice, have published well-authenticated results of such a kind as should satisfy the most sceptical person that its powers are of a very extraordinary order; and, what is of more importance, that it will remove some of the most afflicting forms of disease, when all other known means have either failed, or when they cannot be resorted to with the slightest hope of advantage from them. We often, in the treatment of a disease, know not how far spontaneous efforts in the system have contributed to effect the salutary changes which we witness during the administration of our remedies, and we may frequently doubt whether or not the latter have had any share in producing such results; but these doubts cannot be entertained respecting the greater part of those consequent on the remedy under consideration, in the principal part of the cases above alluded to: for, when we see a person who has been unable to move a limb at all, or not without extreme pain, for several months, perform the natural actions of it with comparative ease and facility after the application of "compression and percussion" to it for a few minutes, it is not possible to avoid believing that it is these means which have effected the change. Yet such a remedy is almost totally neglected by the generality of practitioners, although several years have elapsed since sufficient evidence of its utility was promulgated. Dr. Balfour, however, has sufficient enthusiasm and resolution to incite him to pursue, steadily and vigorously, the good cause he has undertaken, in spite of the difficulties which passion, prejudice, and apathy, have hitherto opposed to it. But he has, perhaps, not acted in the most prudent manner, in replying to, or noticing in any way, the indirect insinuations, malignant misrepresentations, and calumnies, which have been aimed at him and his practice. He should regard them as the natural outcries of impotent malice and vexation; and he may feel assured that no person whose opinion is worthy of consideration will fail, sooner or later, to appreciate them as they deserve. We think he violates what is due to himself in noticing them at all: certainly, the showing of indignation is very unbecoming in him, and the manifestation of anger is still more improper. Men who are weak, and mean, and malicious, will be vexed, and lie, and calumniate; but they must not be censured because they expose their evil dispositions,—they can't help it. On such occasions as these, a wholesome lesson may be taken from the gray *houyhnhm*, *Gulliver's* master, who, after having enumerated all the natural vices of the *yahoos*, said, "Yet he no more blamed them for their odious qualities, than he did a bird of prey for its cruelty, or a sharp stone for cutting his hoof." What seems to have hurt Dr. Balfour most, is a feebly-insinuated imputation of *empiricism:* but how can that conduct be termed empirical which evinces a desire to explain and teach all the means of the practice employed, and which has explained them sufficiently well to enable any person of common ability to use them with success? We turn away from these absurdities: it is enough for us to indicate that they have had their influence in preventing a fair trial of the efficacy of the practice; and to show that other causes than the failure of it to effect what we might have been led to expect from it, have prevented it from being generally resorted to.

This pamphlet comprises histories of fourteen new cases, including that published by Dr. Grattan in the first volume of the Transactions of the Dublin College of Physicians. The first was a case of very severe rheumatism affecting the right thigh of a woman 26 years of age, and which had obliged the patient to keep her bed for two months previous to the time when Dr.

Balfour was consulted. Dr. Poole, of Edinburgh, had been attending her for six weeks, and had, as he stated to Dr. Balfour, "prescribed in succession all the remedies he had ever read or heard of as beneficial in rheumatism, without the patient deriving the slightest advantage from any of them, with the exception of her appetite being somewhat improved."

"The patient was compelled to lie night and day upon her back, with the limb extended and equably supported on the bed. In no other position could she suffer it for a moment, without the most exquisite pain; every attempt to move it occasioned the most frightful screams. It is impossible, indeed, to conceive a human being in a more distressed and helpless condition than was this woman. There was no external appearance of disease in the limb, but the very idea of any thing touching it was intolerable to the patient. Notwithstanding, I was convinced, from ample experience, that nothing but compression and percussion could be of avail. I proceeded therefore, under the regulation of the patient's feelings, to apply the former, first with my hands, and afterwards with a bandage. I beg leave here to state that, as friction was totally inadmissible in this case, so my practice, in the cure of rheumatism and complaints allied to it, is quite different, not only in principle, but in the mode of application, from that of Mr. Grosvenor of Oxford. I have, moreover, had patients who had been previously under his care, and who say the same thing.

"At first, I proceeded with so much caution and delicacy, that three-quarters of an hour were required to go through the operation every day. For the first eight or ten days little progress seemed to be made. An evident amendment, however, was now observable. The parts could be handled with much more freedom. I now applied percussion to the sole of the foot, in order to give a tremulous motion to the whole of the limb. This accelerated the cure greatly. At the end of a fortnight I began to lift the limb from the bed, and to bend and extend it alternately. Formerly, when the limb was in any degree elevated, the whole limb was seized with tremor, attended with agonizing pain: I instantly checked these tremors, by drawing a bandage very tight round the ankle. This may appear extraordinary, but it is not the less true. Tremor of the inferior extremities can be checked at any time, by grasping firmly the tendon of the heel. The cure now went on rapidly; and, within a month from the time I was called in, the patient was on her legs."

Dr. Poole continued to attend with Dr. Balfour during the treatment above designated, and, on its termination, addressed a letter to the latter, in which, after having described the extreme severity of the disease in the strongest terms, he, on speaking of its favourable issue, says, it was, "to my unqualified conviction, justly attributable to the scientific, safe, and obviously efficacious, operation which happily superseded my practice."

The second case was one of rheumatism affecting the muscles of the arms, of, probably, about three months' duration. The patient, the captain of a passage-smack, was so debilitated that he could hardly walk ; and he had lost in the last three months "forty-five cups of blood." He could "neither put on nor take off a stitch of his own clothes." "I applied compression and percussion," says Dr. Balfour, "to the parts affected for a short time, when he felt so much relieved that he put on his clothes without aid, with the exception of the last throw necessary to send the arms home into the coat. Next day he brought his steward with him to receive instructions, as he was determined to accompany his vessel to London the day after." On meeting accidentally with this patient somewhat more than two years afterwards, he told Dr. Balfour that "his directions were implicitly followed by his steward; that the pain and rigidity of his back, shoulders, and arms, were soon removed ; that he was quickly restored to the perfect and permanent use of them; and that, with the exception of a trifling flying pain occasionally, which he could easily check, the disease had never returned." The author met, on the same occasion, with Mr. Simpson, surgeon to the 56th regiment, who told him that, on his book on Rheumatism having been sent to Malta, where he was, by the direction of the Army Medical Board, he, after perusing it, determined to give the practice it inculcated a trial as soon as opportunities permitted. "I did not need to wait long," he added : "cases of both rheumatism and sprains occurred ; all of which I treated according to his [Dr. Balfour's] method, with immediate and perfect success. My professional brethren were astonished at the rapidity of the cures ; and the practice received the particular approbation of Dr. Warren, inspector of hospitals."

We shall transcribe nearly in detail another case, chiefly because it shows the benefits of the same practice in a form of disease of which we have not yet spoken. It is that of "Miss L—, a young lady of a fine form and stature," who "was seized in the summer of 1817, when residing in the country, with a numbness and partial loss of power of the left inferior extremity, accompanied at times with a prickly sort of pain which frequently attends paralytic affections. Having failed of obtaining relief in the country, she came to Edinburgh for further advice." Her friends advised her to put herself under the care of Dr. Balfour; but this was resisted by a relation, who was prejudiced against him by the family-surgeon. But, as the patient derived no benefit from the measures employed, she determined to have the opinion and aid of Dr. Balfour; whose narrative of the subsequent history of the case we shall transcribe.

"The disease consisted in a partial loss of sensation and power in the left inferior extremity, from the middle and back part of the thigh downwards. The patient could walk, but without confidence. In throwing her weight on the limb, she was always afraid of coming down; and her foot could not grasp the ground. There was no pain in the limb, unless a prickly sensation, to the extent of about a hand-breadth in the thigh where the disease commenced, might be called pain. This prickly sensation was excited by firmly grasping the part.

"I told my patient that percussion was the only remedy from which I could expect any benefit in her case; and that it must be applied daily, and continued till a cure should be obtained. She assented, delighted with the prospect of being cured by any means. I proceeded to the operation; and the first application diffused the nervous power through the whole limb. A mixed sensation of pleasure and pain darted through it to the very point of the great toe. From that moment I entertained not a doubt that I would be able to effect a cure; but I warned my patient to expect that it would be very gradually accomplished. I continued my attendance every day for a short time, applying percussion over the whole limb and sole of the foot. This I did on the principle I have often mentioned, that, wherever a stimulus is applied, to that place there is an afflux of blood and nervous energy. If this principle is admitted, and it is self-evident, the objection raised by some writers to the application of percussion in cases of lameness and debility of the extremities from gout, must fall to the ground. Percussion must be admitted to be stimulant, and therefore cannot be repellent. No application can attract and repel at the same time. Those, therefore, who attempt to frighten gouty patients from the use of percussion, as calculated to repel the disease to internal parts, have other objects in view than truth or the welfare of their patients. Such writers merely cavil,—they do not reason; and, instead of instructing, only betray their own ignorance.

"In the course of ten days, an improvement was perceptible in my patient's walk. She trusted her weight with more confidence on the limb, and planted her foot with greater firmness. And, having by this time learned to apply the remedy equally well as myself, I left her in the conviction of ultimately recovering the full power of her limb.

"A short time after I left off visiting her, she returned to the country, and I neither saw her nor heard of her for about twelve months. Standing in the middle of the street one day, in conversation with other two gentlemen, I observed a young lady crossing from the pavement towards us, with a firm and equal pace, smiling as she approached, and holding out her hand to me. I gave her my hand, but did not recognize her till she spoke. As her whole manner indicated happiness, I was not afraid to say, 'And how are you?' 'Perfectly well!' she replied with emphasis. 'Did you continue the operation of percussion?' 'Every day since I saw you; and I have used no other remedy whatever.'"

There are hardly any grounds for choice amongst the rest of the cases related in this pamphlet, in respect to the manner in which they render manifest the extraordinary and peculiar efficacy of Dr. Balfour's practice; and we have, we think, adduced what is sufficient to elicit the attention of our readers to the whole.

